# Understanding the Performance of Plant Protein Concentrates as Partial Meat Substitutes in Hybrid Meat Emulsions

**DOI:** 10.3390/foods11213311

**Published:** 2022-10-22

**Authors:** Mirian dos Santos, Daniela Almeida Vieira Fogaça da Rocha, Oigres Daniel Bernardinelli, Fernando Divino Oliveira Júnior, Diógenes Gomes de Sousa, Edvaldo Sabadini, Rosiane Lopes da Cunha, Marco Antonio Trindade, Marise Aparecida Rodrigues Pollonio

**Affiliations:** 1 Faculdade de Engenharia de Alimentos, Universidade Estadual de Campinas (Unicamp), Cidade Universitária Zeferino Vaz, Campinas, 13083-862, SP, Brazil; 2 Instituto de Química, Universidade Estadual de Campinas (Unicamp), Cidade Universitária Zeferino Vaz, Campinas 13083-862, SP, Brazil; 3 Departamento de Física, Universidade Federal de São Carlos (UFSCar), São Carlos 13565-905, SP, Brazil; 4 Faculdade de Zootecnia e Engenharia de Alimentos, Universidade de São Paulo, Avenida Duque de Caxias Norte 225, Jardim Elite, Pirassununga 13635-900, SP, Brazil

**Keywords:** reduced-meat products, functional properties of plant proteins, in vitro protein digestibility, sunflower protein, rice protein

## Abstract

Hybrid meat products are an excellent strategy to incorporate plant proteins into traditional meat formulations considering recent market trends focusing on the partial reduction in red meat content. In this work, we evaluated the effects of different concentrated plant proteins (soy, pea, fava bean, rice, and sunflower) in partially replacing meat in meat emulsion model systems. Soy, pea, and sunflower proteins showed great compatibility with the meat matrix, giving excellent emulsion stability and a cohesive protein network with good fat distribution. Otherwise, adding rice and fava bean proteins resulted in poor emulsion stability. Color parameters were affected by the intrinsic color of plant proteins and due to the reduction in myoglobin content. Both viscoelastic moduli, G′ and G″ decreased with the incorporation of plant proteins, especially for rice and fava bean. The temperature sweep showed that myosin denaturation was the dominant effect on the G′ increase. The water mobility was affected by plant proteins and the proportion between immobilized and intermyofibrillar water was quite different among treatments, especially those with fava bean and rice proteins. In vitro protein digestibility was lower for hybrid meat emulsion elaborated with rice protein. It is concluded that soy, pea, and mainly sunflower proteins have suitable compatibility with the meat matrix in emulsified products.

## 1. Introduction

A new market trend is emerging with considerable growth in the demand for meat analogous and meat-reduced products for vegan, vegetarian, and flexitarian consumers, individuals concerned about their health and well-being, the preservation of the environment, and animal welfare. However, changing from a meat-centric diet to vegetarianism or veganism may be a challenge due to drastic changes in the diet and cultural and social habits, in general, subtle changes in eating habits can be more effective [1], which confirms the increase in flexitarian consumers who represented 42% of the world population compared to 4% vegans and 6% vegetarians, with young people being more adept at reducing meat [2].

For flexitarians, hybrid meat products are an interesting alternative to reducing meat consumption [3]. Hybrid meat products are made by partially replacing meat with other sustainable protein sources [4], that can add important nutrients to maintain a healthy diet, such as dietary fibers, minerals, vitamins, and bioactive compounds. In addition, meat is a relevant source of nutrients, such as high-biological value proteins, B vitamins, and minerals [5], so combining meat and non-meat protein sources is an interesting approach. Several studies have shown different meat replacers and levels of substitution in hybrid meat products: for instance, partial substitution of pork meat (20%) by extruded pea protein in hybrid sausages [6]; pork meat (30%) by extruded pea, sunflower, and pumpkin proteins in hybrid meatballs [7]; chicken meat (40%) by a mix of plant proteins (soy protein isolate, gluten, and chickpea flour) in chicken sausages [8]; lean meat (22–44%) by a mix of pigeon pea flour, cornflour, walnut, and sesame paste in sausages [9]. In general, hybrid meat products had better cooking yield and less shrinkage during heat than control formulations with 100% meat, however, the texture and sensory attributes are the main parameters changed; usually high levels of meat substitution cause negative effects on sensory attributes, mainly flavor and texture [8,10,11,12,13]. The choice of meat substitute is important from a technological point of view, in general, flours and legume pastes cause greater texture changes due to the high content of carbohydrates, especially fibers that can hinder the creation of a strong protein network; however, the aggregation of different nutrients such as carbohydrates, minerals, vitamins, and bioactive compounds is an advantage of these strategies. The use of concentrated proteins, on the other hand, supports the formation of a denser protein network that may have a greater resemblance to the traditional meat product, and the choice of commercial proteins favors standardization and industrial applicability. Regarding meat substitutes, plant proteins are highlighted due to the wide variety of sources, for instance, legumes, oilseeds, and cereals that could be less expensive than meat [14].

Soy protein is widely used as an extender in meat products due to its functional properties, such as gelling, emulsifying, and water and oil holding capacity. This protein can promote better emulsion stability, cooking yield, texture parameters, and a reduction in shrinkage in meat products [15,16]. Soy is composed of four protein categories based on their sedimentation coefficients (2S, 7S, 11S, and 15S); β-conglycinin (7S) occurs as a trimer and glycinin (11S) occurs as a hexamer, both represent more than 80% of soy proteins [17].

Pea, likewise, is composed of salt-soluble globulins (legumin, vicilin, and convicilin) and water-soluble albumins. Pea protein has a well-balanced amino acid profile, containing a high amount of lysine, leucine, and phenylalanine, but less methionine and cysteine, such as other legumes. Pea protein has been widely used as a substitute for soy or animal proteins in various functional applications because of its availability, low cost, and nutritional value [18]. Fava bean is a rich source of proteins, fiber, and other non-nutrient compounds considered beneficial for health. Vicilin (7S) and legumin (11S) are the major protein portions in fava bean, followed by albumins, prolamins, and glutelins [19].

Rice is the predominant staple food of more than 3 billion people in approximately 100 countries worldwide [20]. Rice protein can be obtained from rice endosperm and rice bran, both by-products of processing raw rice into milled edible rice (white rice). It is hypoallergenic, and nutritious, which gives high potential in the food ingredient industry, especially in gluten-free preparations and infant formulas. Rice has four protein fractions: glutelin (83–86%), globulin (9–11%), albumin (approximately 4%), and prolamin (1–2%) (Doury et al., 2018). Both rice globulin and glutelin are composed of two subunits. Glutelin has poor solubility in water, however, it increases in acidic and alkali conditions [21].

Among oilseed proteins, sunflower protein has attracted interest due to its wide availability, minimal anti-nutritive compounds, and low cost [22]. Sunflower has two main protein types: water-soluble albumins (2S) and salt-soluble globulins (helianthinin, 11S). These two types are present in a ratio of 2:1 (11S:2S, respectively), the seed has 60–80% helianthinins and 25–30% sunflower albumins [22,23]. The 11S helianthinin is similar to legumin in soy and pea, it occurs at hexamer in pH close to neutral, and shows higher resistance to heat denaturation [24]. Sunflower protein has all the essential amino acids, although a lower content of lysine and methionine than recommended by the FAO pattern of essential amino acid requirements [25].

The large difference between plant and meat proteins and the dependence on pH, temperature, ionic strength, and processing treatments used in their extraction (pH changing, heating, drying) exert an influence on their functional properties, such as water and oil holding capacity and emulsifying and gelling abilities [26]. In this sense, investigating the combination of meat and plant proteins is relevant to formulating suitable hybrid meat products with consistent technological and nutritional appeals. Therefore, this study aimed to evaluate the performance of concentrated plant proteins as partial meat substitutes in hybrid meat emulsion model systems regarding their technological issues and in vitro protein digestibility.

## 2. Materials and Methods

### 2.1. Ingredients

The bovine meat (M. gluteus biceps—outside flat cut) and pork back fat were obtained from a local market (Campinas, SP, Brazil). Lean grounded meat (24.0 ± 0.09 protein; 1.41 ± 0.3 fat; pH 5.88 ± 0.03) and pork back fat (9.13 ± 0.09 protein; 81.4 ± 0.57 fat; pH 6.13) were packed in a vacuum bag and stored at −20 °C in a conventional freezer model CHB42DBANA (Consul, Joinville, Brazil) until use. Soy protein concentrate—SPC (Seara Foods, Osasco, Brazil), Pea protein concentrate—PPC (Seara Foods, Osasco, Brazil), Rice protein concentrate—RPC (Rice Pro 80, Grankow, Joinville, SC, Brazil), and Fava bean protein concentrate—FBPC (Vitessence Pulse 3600,Ingredion, Mogi-Guaçu, Brazil) were kindly donated by the respective companies. Sunflower protein concentrate (SFPC) was purchased from a local market (Zona do Cerealista, São Paulo, Brazil). Sodium erythorbate and sodium nitrite were donated by Kerry (Campinas, SP, Brazil).

### 2.2. Experimental Design and Hybrid Meat Emulsions Preparation

Six meat emulsion treatments were elaborated in three batches made on different days, as shown in Table 1. The meat emulsions were elaborated in a food processor (Mixer IBE20, Electrolux, Brazil). Meat, hydrated plant proteins, NaCl, sodium nitrite, and part of the ice were processed until complete homogenization. Sodium erythorbate, pork fat, and the remaining ice were incorporated and homogenized until the temperature reached 12–14 °C. The hybrid meat emulsions (approximately 35 g) were inserted into plastic tubes (50 mL) and centrifuged (Excelsa^®^ II 206-BL, Fanem^®^, São Paulo, Brazil) at 1225 G for 1 min to remove the air and cooked in a water bath (from 35 to 80 °C, approximately 45 min). After cooking, the samples were cooled in a water-ice bath (1 °C) and immediately refrigerated at 4 °C.

### 2.3. Preparation and Physicochemical Characterization of Plant Proteins

The selected plant proteins were previously analyzed for proximate composition, pH, and color (whiteness). Immediately before the process, these plant proteins were pre-hydrated in cold water (4 °C), considering obtaining the final concentration of 20 g/100 g of protein based on their initial crude protein contents (Table 2).

### 2.4. Proximate Analysis, Emulsion Stability, Color, Aw, and pH

Moisture and protein content were determined according to the methodology described by the Association of Official Analytical Chemists [27]. Lipid content was measured according to the cold lipid extraction methodology [28]. Proximate composition was evaluated in two repetitions per batch of each treatment. Emulsion stability was evaluated as the difference between the initial weight of the sample before cooking and the weight after removing the exudate released in the cooking process and expressed as the percentage of exudate and dry losses, the analysis was performed in two repetitions per batch. The pH of the meat emulsions was evaluated at room temperature (24–25 °C) using a HI 98163 meat pH meter (Hanna Instruments, Nuşfalău, Romania) associated with a penetration probe in three repetitions per batch. For instrumental color and water activity (aw), samples were ground using an MPR871 food processor (Oster, Ningbo, China). Aw was measured on the Aqualab water activity meter (Decagon, Pullman, USA) in two repetitions per batch. Instrumental color was determined on a CM-5 spectrophotometer (Konica Minolta, Tokyo, Japan), operating with D65 illuminant, 10° observation angle, SCE mode (relative to sample brightness), using the CIELab color system. Whiteness (W) was determined with the following formula: 100 − [ (100 − L*)2 + a*2 + b*2 ] ½ [29]. Color analysis was performed on three repetitions per batch of each treatment.

### 2.5. Water Holding Capacity (WHC)

The water holding capacity (WHC) of hybrid meat emulsions was determined according to the adapted pressed juice methodology [30], as follows: the samples (approximately 1 cm^2^) were weighed on two sheets of filter paper (Whatman nº1) previously stored in a desiccator for 12 h. Samples were compressed with a cylindrical probe (Ø 50 mm) for 30 s at 78.45 N using a TA-XT2i Texturometer (Texture Technologies Corp., Scarsdale, USA) with a load cell of 25 kg, and trigger force of 5 g. After compression, the sample was removed from the filter paper that was immediately reweighed. The percentage of fluid weight lost from the sample was quantified and correlated with WHC. The analysis was performed in three repetitions per batch of each treatment.

### 2.6. Texture Profile Analysis

Texture profile analysis was evaluated at room temperature (25–27 °C) in a TA-xT2i Texture Analyzer (Texture Technologies Corp., Scarsdale, NY, USA) with a load cell of 25 kg, and with a trigger force of 5 g. Six samples (2 cm height and approximately 2.2 cm diameter) per batch of each treatment were axially compressed into two consecutive cycles, with an interval of 5 s and 50% of compression, using a cylindrical probe (Ø 45 mm) at a constant speed of 1 mm/s. Data were analyzed for hardness (N), springiness (mm), cohesiveness (dimensionless), and resilience (dimensionless).

### 2.7. Rheological Oscillatory Measurements

The viscoelastic behavior of meat emulsions was measured by small amplitude oscillatory measurements using a stress-controlled rheometer Physica MCR 301 (Anton Paar, Graz, Austria). A frequency sweep from 0.1 to 10 Hz was performed at a temperature of 4 °C with a fixed strain rate of 1% using a rough parallel plate (4 cm in diameter) with a gap of 1.5 mm. A temperature sweep from 25 to 90 °C, rate of 5 °C.min^−1^, was performed at 1.0 Hz and 1% strain. To avoid water loss during heating, a thin layer of mineral oil was added around the parallel plate. Elastic component (G′) and viscous component (G″) were recorded. All measurements were performed in triplicate.

### 2.8. Low-Field NMR Spin-Spin Relaxation (T2) Analysis

Water mobility was analyzed by measuring the NMR T2 relaxation time performed on a Bruker Minispec MQ20 NMR analyzer (Bruker BioSpin, Rheinstetten, Germany) operating at a frequency of 20 MHz and 20 °C. The spin-spin relaxation time, T2, was measured using the Carr-Purcell-Meiboom-Gill (CPMG) sequence, with 90° and 180° proton pulses of 8.5 and 16.6 ms, respectively, and an echo time of 160 ms. Data were acquired from 20,000 echoes with 16 scan repetitions. The repetition time between two successive scans was 15 s. T2 relaxation decays were evaluated by distributed exponential fit analysis using Peakfit software (version 4.0, Systat Software, Chicago, USA) by inverse Laplace transform algorithm. The areas and relaxation times of two distinct water populations (T2a and T2b) were determined by cumulative integration using log-normal distribution. The analysis was carried out in three replicates, one per batch.

### 2.9. Microstructure Analysis

The microstructure of freeze-dried samples (0.3 cm^2^ and 0.1 cm height) was evaluated in a TM 4000 tabletop scanning electron microscope (Hitachi Technologies, Tokio, Japan) with an accelerating voltage of 10 kV, emission current of 52,000 nA, a vacuum of 50, and lens mode 4. The images were taken at a magnification of 250× and a scale bar of 200 µm.

### 2.10. Confocal Laser Scanning Microscopy

The analysis was evaluated using a Leica TCS SP5II microscope (Leica Microsystems Heidelberg, Germany) equipped with a helium/neon laser according to a previously methodology [31]: a thin sample of each treatment was stained with 20 µL of fluorescein isothiocyanate (FTIC) solution (0.02% *w*/*v* in acetone) and 20 µL of Nile red solution (0.02% *w*/*v* in methanol). The sample was carefully inserted into the center of the cell view. Analysis was performed at an interval of 1 h after dye addition using fluorescence excitation of 500–530 nm for FTIC and 505–586 nm for Nile red. Representative area data were obtained for each sample using a 20× magnification objective and a 100 µm scale bar.

### 2.11. In Vitro Protein Digestibility Analysis

The meat emulsions were grounded in a blender model 31BL91 (Waring Commercial, Connecticut, USA) for 10 s and subjected to the INFOGEST in vitro digestion protocol [32], described by a previous study [33] with modifications: samples (5.00 g) were placed in a falcon tube of 50 mL, 5 mL of simulated salivary fluid (SSF) was added and the samples were acclimated to 37 ± 1 °C in a water bath and kept in this condition for 2 min. Immediately afterward, 10 mL of simulated gastric fluid (SGF) and porcine pepsin (25,000 U.mL^−1^, Sigma-Aldrich) were added to the oral bolus, the pH was adjusted to 3.00 with 3 M HCl. The samples were placed in an orbital homogenizer model C-385 (Criemaq, Piracicaba, Brazil) at speed of 30 revolutions/min and kept in an oven (Eletrolab, São Paulo, Brazil) at 37 ± 1 °C for 2 h. After gastric digestion was complete, the simulated intestinal fluid (SIF) 1:1 *v*/*v* and porcine pancreatin (2000 U.mL^−1^ based on trypsin activity, Sigma-Aldrich) were added, the pH was adjusted to 7.00 with 3 M NaOH. The samples were placed in an orbital homogenizer (30 revolutions/min) and kept at 37 + 1 °C for 2 h. After the digestion protocol, digested samples were immediately frozen at −60 °C until analysis. The protein digestibility was determined according to the a previously methodology, with minor modifications: an aliquot of the sample digested was centrifuged at 10,000× *g* at 3 °C for 20 min and 1.0 mL of the supernatant was used for measuring the total nitrogen content (N%) by Kjeldahl analysis. The protein digestibility (PD) was calculated as follows: PD (%) = (D − C)/A × 100, where A is the N% of the sample before digestion, C is the N% of the control with reagents and enzymes without sample, and D is the N% of the digested sample [34].

### 2.12. Statistical Analysis

The experiments were conducted in three independent batches. Data were evaluated for the presence of outliers, normality distribution (Shapiro-Wilk test), and equality of variances (Levene test) at a 95% confidence level (*p* ≤ 0.05). Parametric data were analyzed using the General Linear Model (*p* ≤ 0.05) considering treatment as a fixed effect and replicate as a random effect and Tukey’s post-hoc test (*p* ≤ 0.05). Non-parametric data were evaluated using the Kruskal-Wallis test and Games-Howell post-hoc test (*p* ≤ 0.05) using IBM SPSS Statistics 20 software.

## 3. Results and Discussion

### 3.1. Physicochemical Characterization of Raw Materials and Hybrid Meat Emulsions

The concentrated plant proteins used in the present study were characterized as presented in Table 2. The proximate composition highlighted that the concentrated fava bean protein has the lowest crude protein content, while the others ranged from 74 to 85%. Based on these results, the plant proteins used to formulate hybrid meat emulsions were standardized to achieve a similar protein content to raw meat replacement, as defined in Table 1. This strategy, however, resulted in different water added to the formulations, and also the minor components present in concentrated proteins, such as lipids and carbohydrates, varied among formulations. The effects of these parameters on the functional properties of the hybrid emulsions will be discussed in more depth in the following sections.

The physicochemical parameters of hybrid meat emulsions are presented in Table 3. The moisture content varied among the treatments due to the differences in water used in the plant protein’s hydration and the water lost in the cooking process. FR, FFB, and FC treatments showed the lowest moisture content and emulsion stability. The protein content on the wet basis of hybrid meat emulsions varied from 11.99 to 13.09%, and all of them were lower compared to the control treatment, with 14.31%. The concentrated plant proteins were hydrated based on their protein contents, and it was expected to obtain the same final protein concentration of 20%, however, water loss throughout cooking slightly affected the final protein content in these treatments. The lean meat had higher protein content than hydrated plant proteins, which explained the highest value of protein presented by FC. The lipid content varied in the samples as a function of the fluid loss in the cooking process. Those with lower moisture had higher lipid contents. Also, the higher lipid content of the concentrated rice and pea proteins compared to the others (Table 2) influenced the lipid concentration of the FR and FP treatments.

Emulsion stability (ES) during heat treatment is an essential parameter for evaluating the global quality of meat product formulations since it influences yield, texture, and shelf-life. FSF showed the highest ES, followed by FS and FP; FC FR, and FFB treatments (Table 3). Soy and pea protein presents a great capacity for binding water and fat, in addition to emulsifying properties [15,17,35]. Sunflower protein, in the same way, has shown excellent functional properties, especially fat absorption, emulsifying, and foaming ability [22,23,25]. 11S globulins, the main fraction in these proteins, are recognized as good emulsifiers [24]. Moreover, the incorporation of 2% NaCl (ionic strength 0.34 mol/L) possibly increased protein solubility, which plays an important role in the emulsifying ability since it facilitates the migration of proteins and spreading at the oil-water interface [36]. The pH affects the nature and distribution of the net charge of the protein; generally, the proteins are more soluble in values of pH above and below their isoelectric points (pI) due to increased interaction with water. At pH closer to pI, the protein-protein interaction increases because the electrostatic forces are minimal, favoring protein aggregation [37]. Sunflower 11S helianthinin has a pI close to 7.8 [24], higher than the pI reported for 11S globulins in soy and pea (pH 5–6) [17,35]. The FSF treatment presented a value of pH of 5.97, which possibly increased helianthinin solubility due to a greater distance from its pI. Moreover, the attraction between the positive charges of helianthinin and the negative charges of myosin at pH 6 contributed to a stronger and more cohesive protein interface on the fat particles, reflecting greater emulsion stability for the FSF treatment. On the other hand, rice and fava bean proteins conferred poor emulsion stability to the hybrid meat emulsions. The main fraction of rice protein is glutelin, an alkali-soluble protein that has poor solubility in pH between 2–10, with a minimum close to pH 4.5, its isoelectric point [38], which impacts the emulsifying capacity. A previous study showed the emulsifying properties of rice protein increased significantly at pH > 7 due to the breakdown of disulfide bonds in alkali conditions. In addition, the same authors demonstrated that the increase in NaCl concentration (0.4–2.0%) decreased the emulsifying capacity of rice protein due to the reduction in its solubility [39]. Fava bean protein used in this study had the highest content of carbohydrate fraction, mainly fibers, showing a negative effect on ES. The control treatment also presented lower emulsion stability because of the denaturation of myofibrillar proteins after the heat treatment, which tends to favor water loss. Also, this formulation did not have any co-adjuvant component, such as phosphate or starch, to retain the water released during cooking. The dry-matter loss showed the same behavior as fluid loss, however, FFB treatment demonstrated the highest value, which could be linked to the soluble carbohydrate fractions present in fava bean protein. The WHC ranged from 84.49 to 77.27%, and it was lower in hybrid meat emulsions compared with the control treatment (Table 3). A possible explanation is the softer and weaker product structures in the hybrid meat emulsions compared to FC treatment facilitating the free water loss under compressive forces.

The a_w_ presented a small range among treatments, from 0.9724 to 0.9762. The a_w_ of hybrid meat emulsions was slightly different from the FC. FFB treatment presented the lowest Aw value possibly linked to the presence of low molecular weight carbohydrates in the FBPC. pH is an important parameter for the stability of meat products, as it affects the electric charge of proteins and, consequently, their interaction with water. For emulsified meat products, pH values around 6.00 are ideal for improving the functional properties of myosin, but on the contrary, the closer the pH approaches its isoelectric point (approximately 5.3) lower the interaction between myosin-water. The pH values ranged from 5.79 to 6.18, and the hybrid meat emulsions showed higher or equal (for FR) pH to the FC treatment. These values mainly reflected the intrinsic pHs of the concentrated plant proteins (Table 1). Interestingly, treatments with higher pH values were those with better emulsion stability. Thus, possibly both the effect of plant proteins themselves on binding water and the distance from the isoelectric points of meat and plant proteins contributed to the better emulsion stability of FS, FP, and FSF treatments.

**Figure 1:**
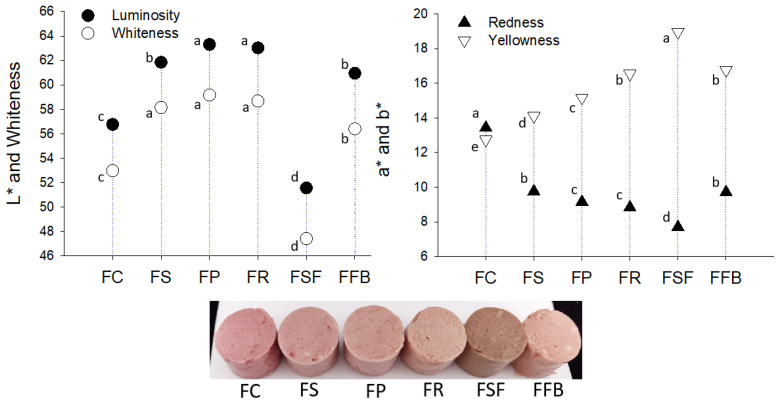
Instrumental color parameters of hybrid meat emulsions elaborated with plant proteins as partial meat substitutes. Mean values with different letters on the column differ from each other (*p* ≤ 0.05) according to the post hoc Tukey’s test (a* and b* coordinates) and Games-Howell’s test (L* coordinate and Whiteness). FC: control; FS: soy; FP: pea; FR: rice; FSF: sunflower; FFB: fava bean.

Color is one of the most important sensory characteristics to guide consumer choice. Cured emulsified products present a pink color due to the formation of the nitrosohemochrome pigment. Thus, the myoglobin content in the formulations and the intrinsic color of each plant protein contributes to the coloration of hybrid meat emulsions. As seen in Figure 1, the red coordinate, a*, as expected, was higher in the FC. Regarding hybrid meat emulsions, the luminosity, L*, and the yellow coordinate, b*, were higher when compared to the FC, except for the FSF treatment, which presented a dark color due to the contribution of SFPC. Based on the color, soy, pea, rice, and fava bean concentrate proteins are appropriate for cured emulsified products that have a characteristic pink color, since this attribute can be easily enhanced with a dye, a strategy widely used in commercial meat products. Sunflower concentrate protein, on the other hand, is suitable for emulsified non-cured cooked products elaborated with red meat, since they usually have a brown color after cooking.

### 3.2. Rheological Parameters of Hybrid Meat Emulsions

**Figure 2:**
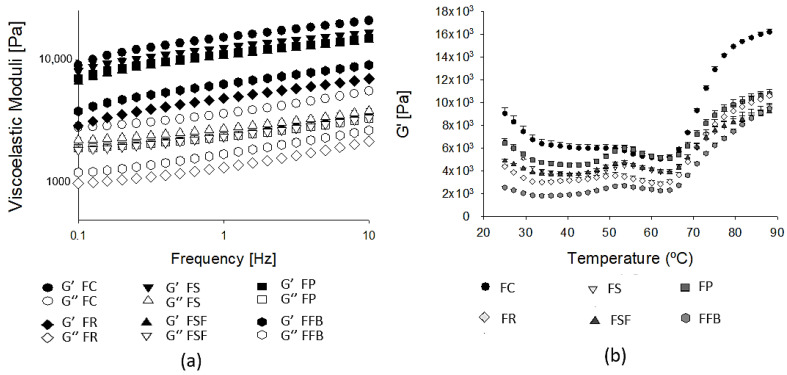
Frequency sweep (**a**) and temperature sweep (**b**) of hybrid meat emulsions elaborated with plant proteins as partial meat substitutes. G′: storage modulus, G″: loss modulus. FC: control; FS: soy; FP: pea; FR: rice; FSF: sunflower; FFB: fava bean.

Figure 2 shows the behavior of storage (G′) and loss modulus (G″) as a function of frequency (Hz). All emulsions showed a gel-like behavior with a prevailing storage modulus (G′ > G″). Both G′ and G″ were only slightly dependent on frequency, which is characteristic of raw meat emulsions [40,41]. FC showed the highest values for G′ and G″, while FR and FFB presented the lowest ones. The water holding capacity of soy, pea, sunflower, rice, and fava bean protein was 15.6 ± 0.9, 8.2 ± 0.1, 7.6 ± 0.5, 3.2 ± 0.2, and 1.9 ± 0.4, respectively. These values can be related to rheological properties since a higher WHC promotes the formation of a more homogeneous structure (protein network) in raw hybrid meat emulsions. Besides, FS, FP, and FSF possibly induced a better dispersion of fat globules based on their emulsion stability, increasing the viscoelastic moduli of the batter, and consequently, G′ and G″ values in these treatments as compared to hybrid meat emulsions FR and FFB.

The effect of heating from 25 to 90 °C on G′ and G″ was similar for all treatments as can be observed in Figure 2. A slight decrease in G′ values was observed between 25 to 45 °C, which was associated with the increase in molecules mobility. A slight increase in G′ was observed around 50 °C, followed by a decrease in the elastic modulus between 52 °C to 65 °C. A decrease in elastic modulus near 50 °C in meat emulsions may be correlated to an increase in protein mobility, rupture of hydrogen bonds, and the unfolding of myosin tails [42]. Above 65 °C, there was a pronounced increase in G′ for all treatments, mainly for FC (only meat proteins) indicating gel formation through cross-links and hydrophobic interaction between protein chains of myosin. As the temperature approached 90 °C, the values of G′ were closer in the hybrid meat emulsions, which demonstrated the main influence of myosin in gelling formation. Plant proteins have suitable functional properties, however, there is a great difference compared to myosin. For instance, these plant proteins are globular instead fibrous, and the main fractions are oligomeric, showing great stability to thermal denaturation. Globulins (7S and 11S) are the main constituents in soy, pea, fava bean, and sunflower proteins. The denaturation temperatures reported for these proteins are higher than myosin: 69–83 °C for 7S and 87–95 °C for 11S globulins in soy, pea, and fava [43,44,45], 100–105 °C for 11S helianthinin in sunflower [23,24], and 82 °C for rice glutelin [46]. Despite both protein sources (animal and vegetable) having their denaturation mechanisms influenced by pH, ionic strength, and heating process, plant proteins are obtained from different extraction and concentration methods that add other parameters that can become their technological application more complex [17,43] and must be understood. Regarding ionic strength, NaCl reinforces the nonspecific charge-shielding effect between charged groups of proteins, reducing inter- and intrachain repulsions, and may also increase the intramolecular hydrophobic interaction [47]. An earlier work demonstrated that 100 mM of NaCl increased the denaturation temperature of pea vicilin and legumin from 70.5 to 82.4 °C and from 77.2 to 87.5 °C, respectively [48]. pH, also influences the denaturation of proteins, since proteins usually presented the highest thermal stability in pH close to pI [24]. Considering that the average temperature of cooked meat products is close to 75 to 80 °C, most plant proteins will probably show an unnoticed denaturation process, which may even decrease the texture parameters of meat products. A recent study reported a reduction of the G′ values and texture parameters in hybrid sausages with partial substitution of pork meat by pea extruded protein, correlating to the inadequate unfolding of pea proteins, the occurrence of protein aggregates, and the less binding possibilities within the meat matrix [6].

### 3.3. Texture Parameters of Hybrid Meat Emulsions

**Figure 3:**
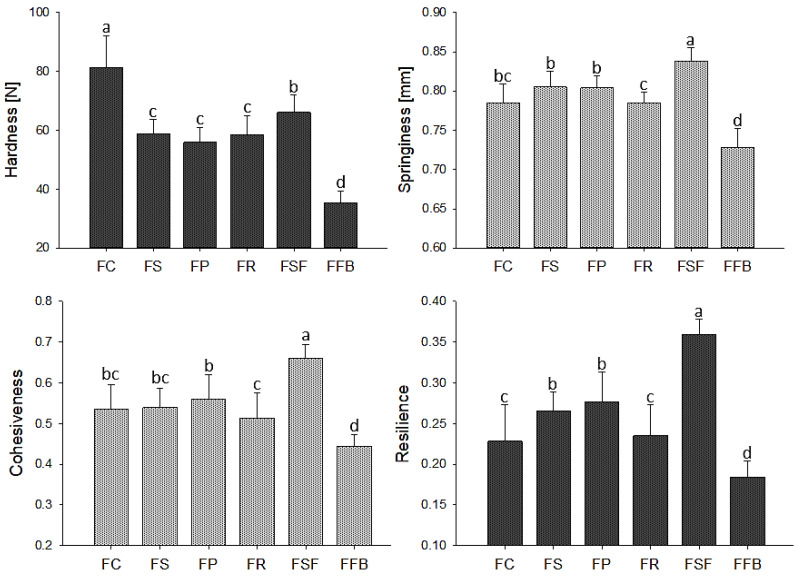
Mean values for texture parameters of hybrid meat emulsions elaborated with plant proteins as partial meat substitutes. FC: control; FS: soy; FP: pea; FR: rice; FSF: sunflower; FFB: fava bean. Mean values with different letters on the columns of each parameter differ from each other (*p* ≤ 0.05) according to the post hoc Tukey’s test.

**Figure 4:**
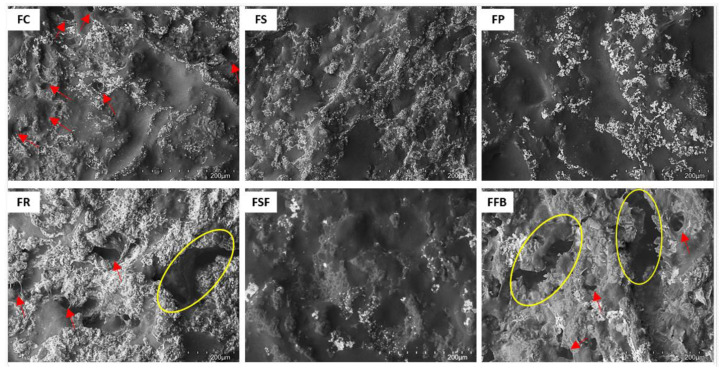
Microstructure images (magnification 250×, scale bar 300 µm) of hybrid meat emulsions elaborated with plant proteins as partial meat substitutes. FC: control; FS: soy; FP: pea; FR: rice; FSF: sunflower; FFB: fava bean.

Texture parameters showed high variation between treatments (Figure 3). Hardness and resilience were the attributes most affected by the partial meat substitution with plant proteins. FC treatment, as expected, showed the highest value for this parameter due to heat denaturation, followed by protein gelation of myofibrillar proteins, mainly myosin. As aforementioned, it was the main responsible for the increase in hardness in the hybrid meat emulsions since plant proteins had an unnoted contribution to denaturation/gelation throughout cooking (up to 80 °C), corroborated by dynamic rheological analysis from temperature sweep. Treatment FSF presented the highest values for all parameters evaluated (except hardness), which are in agreement with the highest ES. Better fat emulsification contributed to obtaining a cohesive protein matrix that, compared to the other treatments, was more capable to resist and easily recover its original form after stopping the deformation, which is observed in springiness and resilience results. FS and FP treatments showed similar behavior for all texture parameters, also corroborating the ES results. FR showed poor emulsion stability and a highly heterogeneous protein matrix (Figure 4), although FR hardness did not differ from FS and FP treatments that presented great emulsion stability. A possible explanation for these differences could be related to a higher water loss during cooking. In meat emulsions, water loss exerts influence on the texture, since water acts as a plasticizer. In general, meat products with high water loss present an increase in hardness [49]. FFB presented the lowest values for all parameters, which can be related to the higher carbohydrate fraction of the fava bean protein concentrate hampering the protein-protein interaction and resulting in a weak protein network. Similar study showed that carbohydrates from lupine flour prevented the formation of a strong protein matrix, causing a decrease in the texture parameters of hybrid beef sausage [50]. Despite the unnoted contribution of plant proteins in gel formation and, consequently, in obtaining hybrid meat emulsions with a firm texture comparable to control, the interaction between water and proteins and, therefore, water holding during cooking was improved. Furthermore, for hybrid meat emulsions, FSF, FS, and FP, the high values for cohesiveness and springiness indicated that they will have suitable sliceability properties [51], unlike FR and FFB treatments.

### 3.4. Microstructure and Confocal Microscopy of Hybrid Meat Emulsions

The microstructure of meat emulsions (Figure 4) showed a homogeneous and cohesive topography with a high proportion of smooth areas for FS, FP, and FSF treatments. The lipid phase (in white) was homogeneously distributed in these treatments, which is in line with the high emulsion stability shown by them. FC treatment showed a great smooth area; however, with some little holes distributed for the protein matrix that could be linked to the water released during cooking. Otherwise, FR and FFB treatments showed a heterogeneous topography, with large discontinuous fractures along the protein matrix that is related to the lower values for emulsion stability, and rheological and texture parameters shown for these treatments.

**Figure 5:**
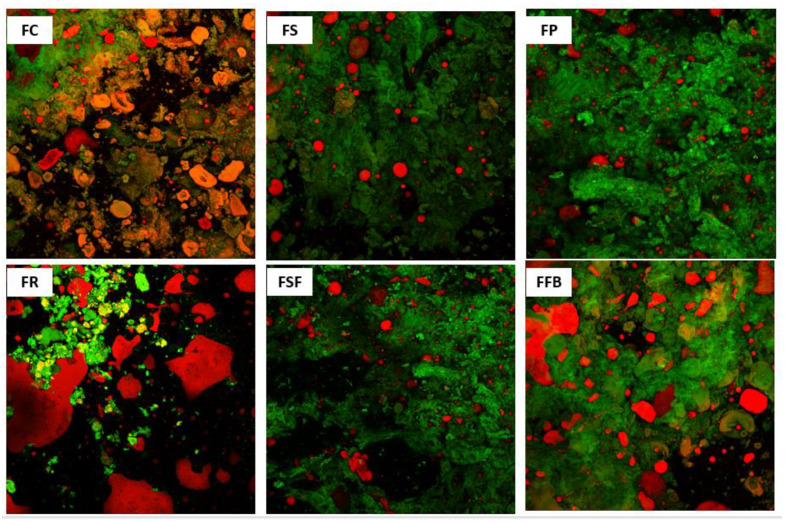
Confocal scanning laser microscopy images (magnification 20×, scale bar 100 µm) of hybrid meat emulsions elaborated with plant proteins as partial meat substitutes. FC: control; FS: soy; FP: pea; FR: rice; FSF: sunflower; FFB: fava bean.

Figure 5 shows the confocal microscopy images of the meat emulsions, where red is dying the dispersed fat, and green is the protein matrix. In general, FC, FS, FP, and FSF treatments showed similar characteristics with the fat dispersed in small and large globules trapped in the protein matrix, this non-homogeneous dispersion of fat globule sizes may be due to a limited comminution process because these are model systems where the processing time was short. Furthermore, due to the high melting point of pork back fat, part of the fat could not be completely emulsified under the experimental conditions applied. In FR and FFB, the lipid phase was concentrated in wide voids, showing substantial coalescence and low interaction between the lipid and protein matrix. Such behavior was more evident in FR treatment, where the lipid phase was on the surface, covering the protein matrix. Both rice and fava bean concentrate proteins showed a weak capacity for emulsification compared to the other plant proteins combined with meat.

### 3.5. Low-Field NMR Spin-Spin Relaxation

**Figure 6:**
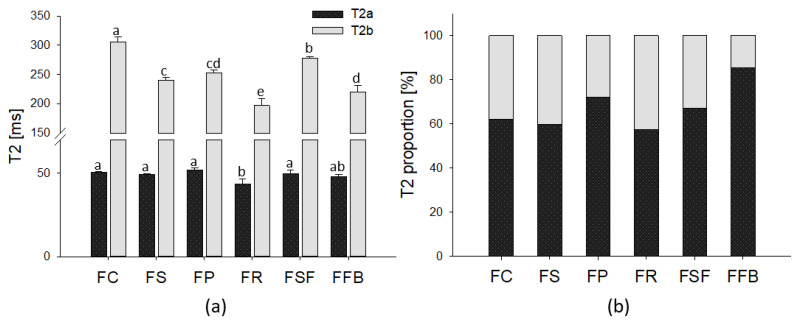
(**a**) The T2 center relaxation time and (**b**) relative area of the two water populations (T2a and T2b) of hybrid meat emulsions elaborated with plant proteins as partial meat substitutes. Mean values with different letters on the columns of each parameter differ from each other (*p* ≤ 0.05) according to the post hoc Tukey’s test. FC: control; FS: soy; FP: pea; FR: rice; FSF: sunflower; FFB: fava bean.

Decomposition of the NMR signal for the meat emulsions resulted in two distinct Log-normal distributions (Lnd): T2a and T2b centered between 44–52 ms and 197–305 ms, respectively (Figure 6). The first population (Lnd) is attributed to immobilized water associated with protein structures and the second one refers to the intermyofibrillar water [31]. Regarding the T2a relaxation time, FR showed the lowest value, and no difference among other treatments was observed. FR showed a significant water loss during cooking since NaCl attenuated the electrostatic repulsion and increased the attractive forces (e.g., van der Waals and hydrophobic) between rice glutelin, leading to protein aggregation and release of water [52,53], which could explain the short T2a relaxation time due to concentration of solutes resulting in less water mobility. Otherwise, T2b, related to intermyofibrillar water, showed a great difference among treatments, FC showed the higher relaxation time, 305 ms, followed by FSF; FS and FP; FFB; and FR. The fibrous structure of myofibrillar proteins associated with denaturation (less interaction with water) and the water channels formed into the protein matrix possibly increased the intermyofibrillar water mobility. Regarding hybrid meat emulsions, the relaxation time decreased as a function of water lost in heat treatment, and the concentration of macromolecules could be related to the rigidity of the molecules containing hydrogen. Regarding the proportion of two water populations, T2a was higher for all treatments, especially for the FFB one which had more than 80% immobilized water. Poor emulsion stability with high water loss during cooking, in addition to the carbohydrate content, contributes to raising the immobilized water and corroborates the lower Aw. T2a and T2b areas were higher for FSF and FP, and lower for FC, FS, and FR. These results could be related to the water-binding of the concentrated plant proteins, as well as to the complex interactions of water with the minority contents of carbohydrates and minerals, which could be influencing its binding and mobility.

### 3.6. In Vitro Protein Digestibility

**Figure 7:**
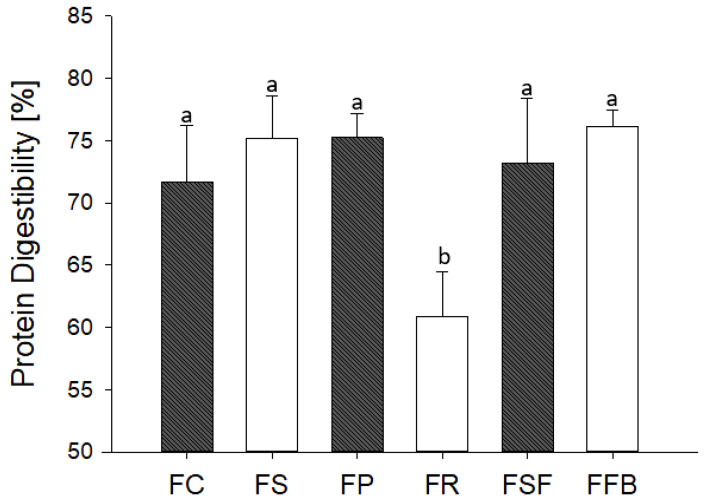
Effects of plant proteins as partial meat substitutes on in vitro protein digestibility of hybrid meat emulsions. Mean values with different letters on the columns of each parameter differ from each other (*p* ≤ 0.05) according to the post hoc Tukey’s test. FC: control; FS: soy; FP: pea; FR: rice; FSF: sunflower; FFB: fava bean.

Protein digestibility greatly varies among foods, being dependent on the protein source, food matrix, the molecular interactions between proteins and other components, and the conditions during food processing and storage [54]. The in vitro protein digestibility based on the total nitrogen content varied from 61% to 76%, in FR and FFB treatments, respectively (Figure 7). Rice protein, as already discussed, could have a low solubility due to the action of NaCl on glutelin that may have contributed to the formation of protein aggregates that difficulted the enzymatic activity. Similarly, the large lipid aggregates due to the poor emulsion stability exhibited in the FR treatment contributed to this result. Despite the large difference between meat and plant proteins, the other treatments did not differ from the control concerning protein digestibility. Plant protein sources, especially legumes, have several anti-nutritional compounds that can interact with proteins and inhibit the accessibility of digestive enzymes and protease inhibitors that prevent their activities [54]. Nevertheless, the plant protein concentrates due to the separation and purification processes show a significant reduction in antinutritional compounds [55] and better digestibility can be achieved [56,57]. Moreover, the modification of protein structures, with different degrees of unfolding, may facilitate the access of enzymes [58].

**Figure 8:**
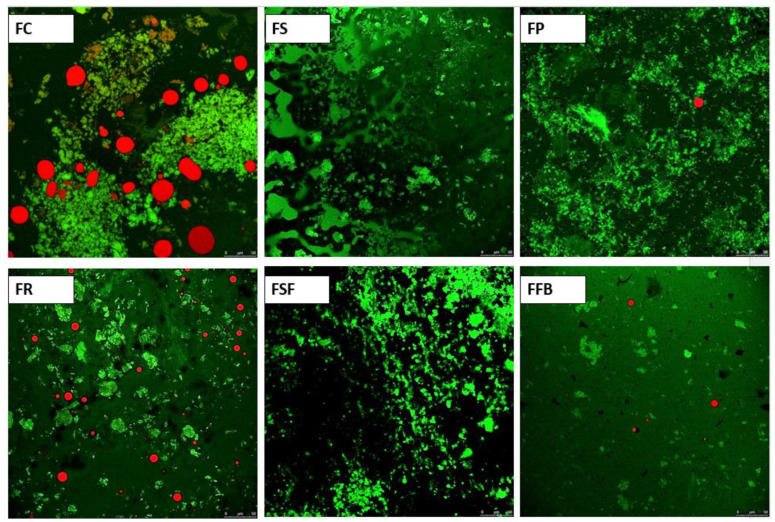
Confocal scanning laser microscopy images of hybrid meat emulsions after in vitro simulated digestibility. Magnification: 40×; Scale bar: 50 µm. FC: control; FS: soy; FP: pea; FR: rice; FSF: sunflower; FFB: fava bean.

The confocal microscopy images of samples after simulated digestion (Figure 8) showed a great number of digested protein fragments among treatments, and several fat globules in FC and FR. FC sample after initial trituration to simulate oral chewing showed bigger pieces in comparison to the other treatments due to its harder texture, which could hinder the enzymatic action during digestion, resulting in a high number of protein fragments and fat globules. A recent study evaluating the effect of time and frequency of chewing on in vitro digestibility of chicken and soy-based vegetarian chicken, demonstrated that smaller bolus fragments increased the degree of protein hydrolysis [59]. FR showed a significant coalescence of fat (Figure 5). These large fat agglomerates possibly difficult the pancreatin action on the lipid phase, resulting in several fat globules at the end of simulated digestion. The rate of lipolysis by gastric lipase was inversely related to the oil globule sizes due to the lower interfacial area of coarse emulsions [60], corroborating our results. Treatments FS, FP, and FS presented few or no apparent fat globules at the end of the simulated digestion. These treatments demonstrated excellent emulsion stability, which indicates that fat was more uniformly distributed in the protein matrix, which possibly facilitated the enzymatic action. The FFB treatment also demonstrated high protein digestibility, which may be related to its very soft texture that allowed it to obtain a paste-like texture with small pieces after initial trituration that may have facilitated digestion, which was in line with a previous study [59]. Based on simulated protein digestibility, the hybrid meat emulsions with plant proteins showed evidence of adequate protein availability, similar to the control treatment. The in vitro digestion of beef steaks incorporated with 4% and 8% of soy protein isolate, rice protein, or lentil flour resulted in a similar distribution of small peptides compared to the control treatment (meat only), which presented more dense and compact protein aggregations in the intestinal phase, all treatments showed a complete disintegration of myofibrillar structure after digestion [59], which demonstrated some evidence of suitable digestibility behavior of meat products elaborated with plant proteins. A previous study, unlike what was observed in the present work, showed the protein hydrolysis for chicken was 89% while for soy-based vegetarian chicken was 70%, this result could be influenced by the extrusion process that generates fibrous structures by cross-linking of proteins, the increased in cross-links contributes to protein aggregation in the vegetarian chicken and can reduce protein susceptibility to enzymatic proteolysis [59]. These results showed the type of process that plant proteins were submitted is a relevant factor to protein hydrolysis.

## 4. Conclusions

Reducing raw meat in meat product formulations is an interesting approach from a sustainability and a nutritional view, since a greater variety of macro- and micronutrients from vegetal and animal sources can be combined. In this study, concentrated plant proteins were evaluated as partial meat substitutes in hybrid meat emulsions. Most physicochemical parameters were affected by plant proteins considering their intrinsic characteristics and the compatibility of the meat matrix. Soy, pea, and sunflower proteins conferred great emulsion stability and a suitable texture profile, which was corroborated by micro-structure and confocal images. Adding rice and fava bean proteins, otherwise, resulted in lower emulsion stability, and rheological and texture parameters. Water mobility was affected by plant proteins, and it could be correlated with emulsion stability among treatments. Fava bean protein due to the higher content of carbohydrates increased the immobilized water. In vitro protein digestibility was lower for treatment with rice protein, possibly because the lipid aggregates formed due to the poor emulsion stability acted as a barrier to enzymatic action. The other hybrid meat emulsions did differ from the control treatment regarding protein digestibility, which is important from a nutritional view. The plant proteins evaluated, soy, pea, and sunflower proteins showed a potential use due to their functional properties when combined with the meat matrix. However, evaluating sensory acceptance is fundamental to indicate the feasibility of hybrid meat emulsions made with these plant proteins as well as the global stability during the shelf life.

## Figures and Tables

**Table 1:** Formulations (g/100 g) of hybrid meat emulsions elaborated with plant proteins as partial meat substitutes.

Ingredients	Treatments					
	FC	FS	FP	FR	FSF	FFB
Lean red meat	65.00	32.50	32.50	32.50	32.50	32.50
Hydrated plant proteins	-	32.50	32.50	32.50	32.50	32.50
Pork backfat	20.00	20.00	20.00	20.00	20.00	20.00
NaCl	2.00	2.00	2.00	2.00	2.00	2.00
Sodium nitrite	0.015	0.015	0.015	0.015	0.015	0.015
Sodium erythorbate	0.05	0.05	0.05	0.05	0.05	0.05
Water	13.0	13.0	13.0	13.0	13.0	13.0

FC: meat emulsion control with 100% meat; FS, FP, FR, FSF, and FFB: hybrid meat emulsions incorporated with hydrated plant proteins (soy, pea, rice, sunflower, and fava bean, respectively) as partial meat substitutes (50%).

**Table 2:** Mean values (standard deviation) for physicochemical results of plant proteins.

Plant Proteins	Moisture (g/100 g)	Protein (g/100 g)	Lipid (g/100 g)	Carbohydrate ^1^ (g/100 g)	pH ^2^	Whiteness
SPC	7.33 (0.2)	85.0 (1.9)	3.51 (0.1)	<1.0	7.85 (<0.1)	76.33 (0.2)
PPC	7.82 (0.1)	74.4 (0.8)	8.91 (0.3)	2.2	7.51 (<0.1)	70.26 (0.1)
RPC	3.90 (0.2)	81.1 (2.0)	9.84 (0.1)	3.0	5.68 (<0.1)	81.88 (0.1)
SFPC	6.14 (0.1)	77.8 (0.7)	3.34 (0.1)	2.0	6.79 (<0.1)	50.51 (<0.1)
FBPC	8.53 (0.5)	56.5 (0.3)	3.12 (0.1)	25.1	6.49 (<0.1)	84.52 (0.2)

Informed by producers ^1^; 1% protein solution in deionized water ^2^.

**Table 3:** Mean values (± standard deviation) of physicochemical parameters of hybrid meat emulsions with plant proteins.

Parameters	Treatments					
	FC	FS	FP	FR	FSF	FFB
Moisture * (g/100 g)	61.23 ^b^ (0.19)	64.17 ^a^ (0.44)	63.66 ^a^ (0.49)	59.76 ^c^ (0.54)	64.29 ^a^ (0.26)	58.85 ^c^ (0.22)
Protein (g/100 g)	14.31 ^a^ (0.13)	12.76 ^bc^ (0.20)	12.33 ^cd^ (0.07)	13.09 ^b^ (0.31)	12.28 ^cd^ (0.42)	11.99 ^d^ (0.30)
Lipid (g/100 g)	17.46 ^b^ (0.39)	15.73 ^d^ (0.35)	16.50 ^c^ (0.36)	18.34 ^a^ (0.15)	15.71 ^d^ (0.12)	18.42 ^a^ (0.30)
ES—liquid loss (%)	11.22 ^a^ (1.20)	4.14 ^b^ (0.34)	3.49 ^b^ (0.65)	12.57 ^a^ (1.16)	1.73 ^c^ (0.66)	11.58 ^a^ (1.14)
ES—dry loss (%)	0.98 ^b^ (0.09)	0.35 ^c^ (0.04)	0.30 ^c^ (0.06)	1.03 ^b^ (0.20)	0.15 ^d^ (0.07)	2.03 ^a^ (0.30)
WHC (%)	84.49 ^a^ (0.55)	80.24 ^b^ (1.69)	79.20 ^bc^ (1.40)	78.00 ^c^ (1.75)	77.93 ^c^ (1.73)	77.27 ^c^ (1.28)
Aw	0.9739 ^b^ (< 0.01)	0.9762 ^a^ (< 0.01)	0.9753 ^a^ (< 0.01)	0.9758 ^a^ (< 0.01)	0.9738 ^b^ (< 0.01)	0.9724 ^c^ (< 0.01)
pH	5.79 ^e^ (0.037)	6.18 ^a^ (0.028)	6.13 ^b^ (0.019)	5.79 ^e^ (0.017)	5.97 ^c^ (0.019)	5.87 ^d^ (0.019)

ES: emulsion stability, WHC: water holding capacity, W: whiteness. Mean values with different letters in the same row for the attributes differ from each other (*p* ≤ 0.05) according to the post hoc Tukey’s test or Games-Howell’s test (*).

## Data Availability

The data presented in this study are available on request from the corresponding authors.
